# Annual Meeting of Wisconsin Medical Society

**Published:** 1848-02

**Authors:** Mason C. Darling, C. B. Chapman

**Affiliations:** Pres’t. pro tem.; Secretary


### ANNUAL MEETING.

The Wisconsin Territorial Medical Society convened at the Capitol, on Tuesday, the 25th of January, A. D. 1848.

On motion of Dr. E. A. Mulford, from Walworth, adjourned to Wednesday, the 27th of February.

Pursuant to adjournment the Society met.

On motion of Dr. H. Clark, from Walworth, Dr. Mason C. Darling was appointed President, *pro tem.*

On motion of Dr. C. B. Chapman, of Madison, Dr. Eliab M. Joslin, from Lake Mills, was elected a permanent member of this society.

On motion of Dr. H. Clark, Dr. F. G. Newell, from Racine, was elected a permanent member of this society.

Dr. John Bristol, of Portage, and Dr. Jesse Moore, of Rock, were by vote excused from attendance, they having rendered a satisfactory excuse for their absence.

The society proceeded to the election of officers for the ensuing year. Whereupon

Dr. John B. Dousman, of Milwaukee, was chosen President, and Dr. L. B. Brainard, of Sheboygan, Vice President.

Dr. C. B. Chapman, of Madison, Recording Secretary, Dr. B. B. Cary, of Racine, Treasurer.

Drs. E. B. Wolcott and L. S. Hewett of Milwaukee, and Dr. O. N. Blanchard, of Racine, Censors.

Dr. Jesse Moore, of Rock, Corresponding Secretary.

On motion of Dr. Clark, voted that five delegates be elected to attend the meeting of the American Medical Association at Baltimore in May next.

The following gentlemen were chosen such delegates, viz: Dr. Mason C. Darling, of Fond du lac; Dr. Jesse Moore, of Rock; Dr. E. B. Wolcott, of Milwaukee; Dr. Henry Clark, of Walworth, and Dr. B. B. Cary, of Racine.

On motion of Dr. Newell, the following was also adopted.

Whereas, pure and unadulterated articles of medicine are all-important to the life, health and safety of the people of the United States; and whereas, impure and adulterated articles of medicine are constantly being imposed upon us, therefore

Resolved, That the Territorial Medical Society of Wisconsin, respond to the call of the New York College of Pharmacy in its organized capacity, in memorializing Congress that a law may be enacted that all imported articles intended for medical purposes, which may appear at the custom houses of the United States, shall be subject to inspection and analysis by two or more competent chemists, and if found to be impure and adulterated, to be confiscated and destroyed.

That the President be requested to deliver an address before the Wisconsin Territorial Medical Society at its next annual meeting.

On motion of Dr. Clark, the society then adjourned.

MASON C. DARLING, *Pres't. pro tem.*

C. B. CHAPMAN, *Secretary.*

—*Madison Argus.*

